# Finite Element Study on the Shear Capacity of Traditional Joints between Walls Made of AAC Masonry Units

**DOI:** 10.3390/ma13184035

**Published:** 2020-09-11

**Authors:** Marcin Kozłowski, Iwona Galman, Radosław Jasiński

**Affiliations:** 1 Department of Structural Engineering, Silesian University of Technology, 44-100 Gliwice, Poland; iwona.galman@polsl.pl; 2 Department of Building Structures and Laboratory of Civil Engineering Faculty, Silesian University of Technology, 44-100 Gliwice, Poland; radoslaw.jasinski@polsl.pl

**Keywords:** masonry, wall, joint, autoclaved aerated concrete, experimental testing, numerical simulations, concrete damage plasticity

## Abstract

This paper presents the development of a numerical model aimed at the simulation of nonlinear behaviour of traditional joints between walls made of autoclaved aerated concrete (AAC) masonry units. Nonlinear behaviour and cracking of AAC and mortar were simulated using the concrete damaged plasticity (CDP) model available in the ABAQUS finite element software. The paper also presents and discusses the results of an experimental campaign involving testing six T-shaped, monosymmetric samples with traditional joints between walls loaded in shear. The results were used to validate the numerical model. The validation confirmed that the model is capable of producing accurate results and predicting the structural behaviour with a reasonably good accuracy in elastic and post-elastic stages. Furthermore, a sensitivity study was conducted, in which the variation of elastic modulus, Poisson’s ratio, tensile strength, compression strength and fracture energy of AAC was investigated. Results showed that the variation of elastic modulus, tensile strength and fracture energy is most critical to the structural behaviour of the model, while variation of the remaining parameters has a negligible effect on the results.

## 1. Introduction

The history of masonry structures is almost as long as the history of human civilization. The first traces of the oldest stone constructions were found in the Middle East and are dated to the period of 9000 BC. An example is a stone wall surrounding the terrace in front of a cave in El-Uad (Israel) [1]. Less than 2000 years later, first small-size ceramic elements were found and described in the literature. Initially, the bricks were not fired but only dried in the sun. Only in the middle of the third millennium Before Christ (BC) in Mesopotamia, due to the invention of the firing process, ceramics gained much greater interest [2]. Thanks to the simplicity of production of the masonry by combining small-size ceramic elements and a binder, masonry structures have remained extremely popular up to now. Over the years, along with the development of civilization and technology, new materials were used to produce masonry units, such as concrete, limestone or aerated concrete.

Nowadays, autoclaved aerated concrete (AAC) blocks with thin joints have become one of the most popular materials for the construction of load-bearing walls, mainly in single-family housing or in-fill walls for concrete superstructures [3]. It is produced by mixing cement, lime and gypsum binders, fillers such as sand, and small amounts of aluminium powder (or paste), which acts as a foaming agent. The density of AAC ranges from 300 to 1000 kg/m^3^, and the compressive strength is from 1.5 to 10 MPa. AAC has been used since the mid-1950s (in Europe, >40% of the construction market) as a material for making masonry structures, such as walls and prefabricated ceiling and lintel elements [4]. AAC also shows very good thermal properties (heat transfer coefficient λ = 0.08–0.20 W/mK). Another advantage of walls made of AAC blocks is simplicity and speed of construction. The blocks are available in large sizes, thanks to which the construction process itself is much faster when compared to, for instance, ceramic bricks. In addition, the blocks are very easy to process; they can be cut, grooved and pierced with ordinary tools, e.g., a hand saw. Unfortunately, like any other material, AAC has weaknesses. The main disadvantage of the AAC wall is its low cracking resistance. The issue becomes dangerous in places of concentrated stress (e.g., joints between walls) that are prone to crack [5].

Limited experimental results on the load-bearing capacity of joints between walls can be found in the literature. One of the few examples is research conducted by Paganoni and D’Ayal [6]. T-shaped specimens were designed and tested to reflect the behaviour of the joint as in a real structure. Results confirmed high effectiveness of steel fasteners for the load-bearing capacity of the joints between walls. Maddaloni et al. [7,8] conducted similar tests. In this case, the effectiveness of innovative clamping anchors (carbon fibre rods wrapped longitudinally and spirally with a stainless-steel mat) was assessed. The tests showed high efficiency of both strengthening methods. Results of the performance of H-shaped walls can be found in [9], where authors found difficult to assess the load-bearing capacity of a single joint of the H-shaped samples due to the asymmetrical cracking pattern of two walls. It is also worth mentioning the research by Corrêa et al. [10] who compared the load-bearing capacity of masonry joints using different techniques. Authors considered a wall with a masonry tie, a steel mesh embedded in a support joint and a steel anchor. Studies have shown that the connection of steel elements is able to take over 60% of the load obtained in the wall with a classic masonry tie. From an analysis of the references in the literature, it can be concluded that, although there are promising results, the research regarding wall joints is limited and not sufficient to describe the structural behaviour of the joints and to formulate some practical construction principles with particular emphasis on new technologies for erecting walls (thin-layer joints, light joints, unfilled head joints, etc.). There is also lack of research on the effectiveness of wall joints using anchors that are available and used commonly on the European market.

Eurocode 6 for the design of masonry structures [11] requires that walls joined mutually perpendicularly or diagonally are to be connected with each other in a such a way to ensure the transfer of vertical and horizontal forces from one wall to another. At the same time, the standard does not provide any design approaches nor principles for checking the load-bearing capacity of the joints. This applies not only to the traditional joints between the walls but also to the joints with structural fasteners. Moreover, the empirical procedures for determining the load-bearing capacity of vertical joints between walls have not been standardised.

The current research is a part of the ongoing research project on the connections of walls made of AAC connected using the most popular fittings available on the market, as well as various types of meshes, mats and other elements that would connect walls with each other in a simple and effective way. Results of the past studies can be found in [12,13,14]. So far, the obtained knowledge and results are promising and encourage further work in the field of describing the work of the joint and the use of new methods of constructing joints, using other connectors, using more steel elements in a given weld and optimising the shape of steel flat bars.

Different techniques such as the finite element method, discrete element method, applied element method and the transfer matrix modelling are used to solve engineering issues [15,16]. For the analysis of the behaviour of wall connections, being the subject of this paper, the finite element method (FEM) is used. It provides great opportunities to analyse structures composed of various materials, e.g., reinforced concrete or masonry [17,18,19]. The use of advanced material models allows analysis of structural load-bearing capacity, damage development [20,21,22,23], changes in stress state and adhesion deformation and reinforcement anchoring [24,25,26]. Despite the development of knowledge and the continuous increase of computing power, it is not yet possible to build a numerical model of a structure enabling a global analysis that includes all physical phenomena. Therefore, even in case of advanced FEM models, some simplifications have to be made. These should be adapted for the planned purpose of calculations and output data and should include information available on the material and the possibilities of the computational systems.

Accurate numerical analysis of multi-material constructions is complex because it requires determination of many material parameters. Assumption of the yield surface requires results of material tests in uniaxial and complex load conditions [27,28,29,30] or samples of unusual shapes [31]. In addition to the criteria of material failure, it is also necessary to specify various parameters describing the phenomena occurring at the interface of the combined materials, e.g., reinforcement and concrete or masonry and mortar. Therefore, a correct model requires the use of special contact elements whose parameters are also adopted on the basis of laboratory tests [28,29].

**Figure 1:**
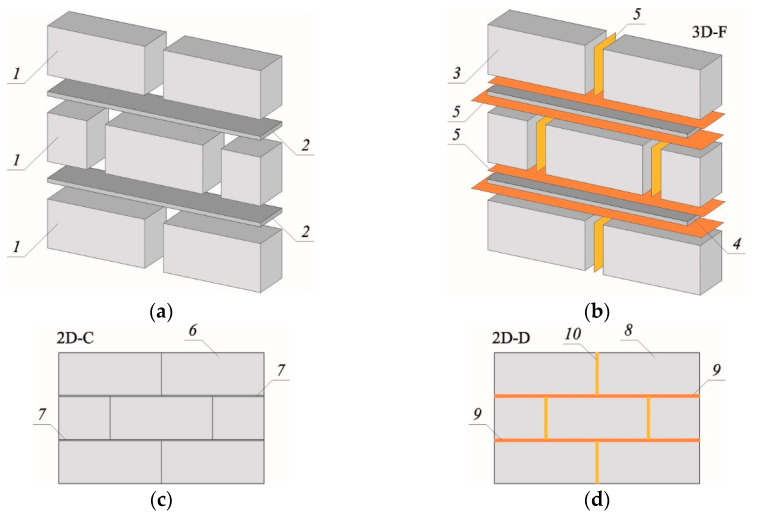

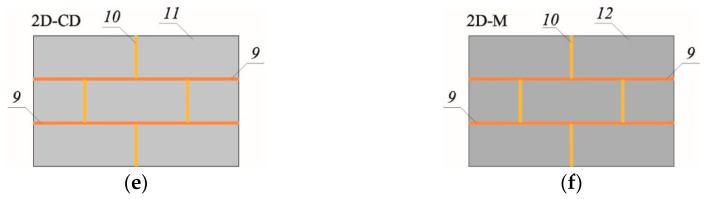
Adaptation of various strategies for building the wall micro-model with thin-wall joints without filled head joints: (**a**) actual structure of the modelled wall, (**b**) spatial wall micro-model (3D-F), (**c**) plane wall micro-model (2D-C), (**d**) plan wall micro-model (2D-D), (**e**) plan wall micro-model (2D-CD), (**e**) plane wall micro-model (2D-M); (**f**) plane wall micro-model (2D-M); 1—masonry element, 2—masonry mortar, 3—masonry element with linear or nonlinear parameters, 4—masonry mortar with linear or nonlinear parameters, 5—contact element of bed and head joints with nonlinear parameters, 6—masonry element with linear or nonlinear parameters, 7—mortar with linear or nonlinear parameters, 8—masonry element with linear parameters, 9—contact element of bed joints with nonlinear parameters, 10—contact element of head joints with nonlinear parameters, 11—masonry element with nonlinear parameters, 12—masonry element with equivalent nonlinear masonry parameters.

For the numerical analysis of walls made of masonry, mainly nonlinear material models are used. These are based on experimental observations and differ in the constitutive relationships and strength criteria. Parameters can be adopted either for all components of a wall (masonry units and mortar) or substitute material that has averaged properties of the components. For this reason, in the practice of modelling masonry structures, three main modelling approaches are distinguished (Figure 1):

- Macro-modelling—The approach treats the masonry structure as a material with averaged mechanical parameters for masonry units and mortar. This model is used in practical calculations of large masonry structures (size-wise finite elements may include even several masonry units) [32,33,34,35];
- Meso-modelling—This is a variation of the macro-model variety that, in an way analogous to the periodic microstructure, includes nonlinear relationships between the average stresses and the average deformations of an element consisting of masonry units and mortar layers that is equivalent to a given body of the same dimensions. In this approach, a basic cell (representative volume element [36]) is established. It contains all geometric and physical information regarding any type of wall component [37,38];
- Micro-model—This treats masonry as a heterogeneous material. Division into finite elements occurs within each material (mortar, masonry unit). Different, nonlinear behaviour of brick and mortar with possible consideration of the interaction between them is used. Such a wall model is commonly used for the analysis of small structures or detailed analysis.

The main aim of the current study was to develop a nonlinear two-dimensional finite element model to simulate the structural behaviour of traditional joints between walls made of AAC masonry units. For FE modelling, a 2D micromodel of the wall without contact elements at the interface between the mortar and masonry elements was used. The paper also presents the results of experiments with full-size samples, which were used to validate the FE model.

## 2. Experimental Observations

### 2.1. Test Specimens and Test Set-Up

The FE study shown in this paper was performed on the basis of past experimental studies presented in [13]. The main aim of the past experimental study was to investigate the shear capacity of traditional, unreinforced joints between walls made of AAC masonry units. In this paper, only major observations and conclusions were presented. Further details of the experimental set-up and test results can be found in [13].

**Figure 2:**
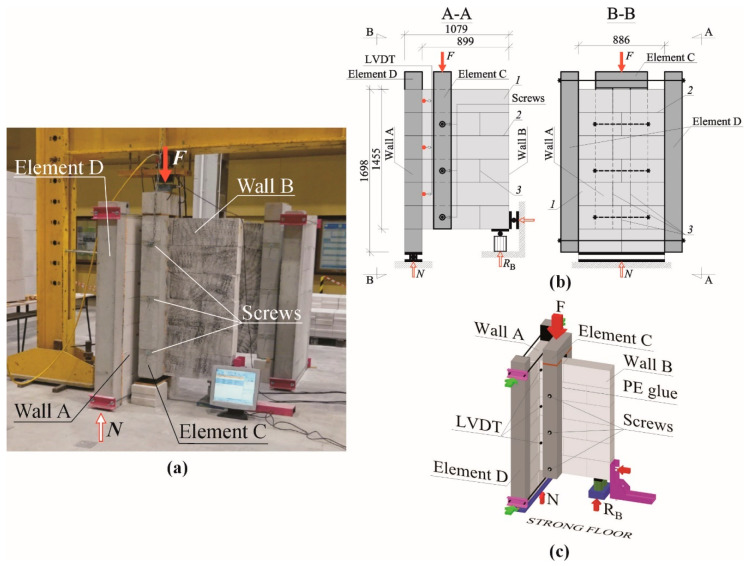
Experimental testing of the shear capacity of traditional joints between walls made of AAC masonry units: (**a**) Test rig in the laboratory; (**b**) Test set-up with detailed description, (**c**) Test set-up isometric view; 1—AAC masonry units, 2—bed joints, 3—head joints.

In the experimental campaign, six T-shaped, monosymmetric samples with traditional joints between walls were built and tested (Figure 2a). The samples are denoted in this paper as P1–P6. Each sample consisted of two perpendicular walls with approximate dimensions of 180 × 886 and 180 × 1079 mm^2^, denoted herein as Wall A and Wall B, respectively (Figure 2b). The walls were raised with AAC blocks with the dimensions of 180 × 240 × 595 mm^3^. In the walls, all bed joints were filled with cement mortar 3 mm in thickness, whereas head joints were left unfilled. In this way, only three blocks of Wall B were penetrating the Wall A and thus actively transferring the shear forces between the walls (Figure 2b,c).

The tests were conducted in a custom-made test set-up designed and manufactured for the purpose of the experiments. The shear force was introduced to Wall B via a reinforced concrete element (Element C, Figure 2b) placed vertically and fixed to Wall B using three steel screws and, additionally, by filling the gap between the concrete element and the wall with polyurethane adhesive to ensure full transfer of shear forces. Wall B was tied with vertical, reinforced concrete elements (Element D, Figure 2) and bonded to the wall with polyurethane glue and steel screws.

The load was introduced to the upper part of the beam via a transverse element by a hydraulic jack with the capacity of 1000 kN with a constant displacement rate of the piston of 0.1 mm/min. All walls were tested monotonically until failure.

Besides measuring the force in the hydraulic jack (force *F*, Figure 2b,c), a reaction under Wall B was registered throughout the test by two load cells (reaction force *R_B_*, Figure 2b). In this way, the vertical reaction under Wall A (reaction force *N*, Figure 2b,c) can be calculated back by subtracting the reaction of Wall B from the total force F in the hydraulic jack as follows:

(1) $$N=F-R_{B}.$$

To measure the relative displacement between Wall A and Wall B, a set of three linear variable differential transformers (LVDTs) were mounted to the samples (three locations are shown in Figure 2b). Fixed points (aluminium angles) were screwed to Wall B, whereas the LVDTs were mounted on the AAC units of Wall B.

During the tests, displacements and deformations were measured for two samples of each series with digital image correlation (DIC) using the ARAMIS 6M system by GOM GmbH, Brunswick, Germany (the class of reading accuracy for displacements was 1%) [39,40,41]. The solution provided complete, three-dimensional, real-time surface, displacement and strain results for multiple measuring positions from the test object surface. Characteristic points (spackle pattern) were applied with black paint before testing. Strains were measured with a high-resolution camera and evaluated as changes in distances between the characteristic points allowing in post-processing stage to capture cracking formation (Figure 4c).

### 2.2. Experimental Results

In the paper, force *N* (being a reaction under wall B, Figure 2b) indicates the load acting on the joint between Wall A and Wall B. The relative displacement between walls was calculated as an average value of three measurement points.

**Figure 3:**
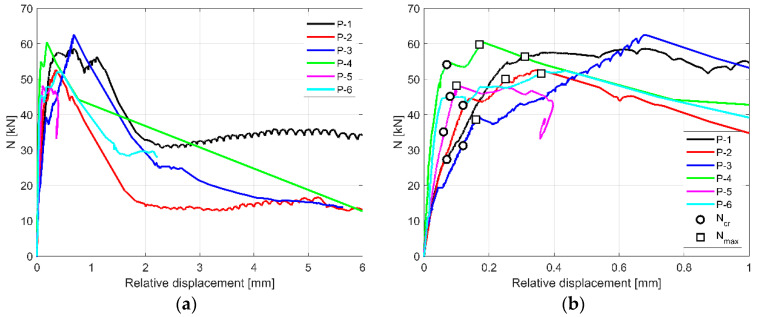
Experimental load-displacement responses for the tested specimens P1–P6: (**a**) full range of relative displacement; (**b**) range of relative displacement up to 1.0 mm.

Figure 3a presents force (*N*)—relative displacement curves of the wall responses for the tested specimens. In addition, in Figure 3b, the behaviour of the samples in the range of relative displacements from 0.0 to 1.0 mm was shown. In the Figure 3b, markers indicating the load at initial cracking (*N_cr_*) of the Wall B and at the maximum load (*N_max_*) are shown. The values (*N_cr_* and *N_max_*) correspond to the relative displacement at initial cracking (*u_cr_*) and maximum load (*u_max_*) in Table 1.

**Figure 4:**
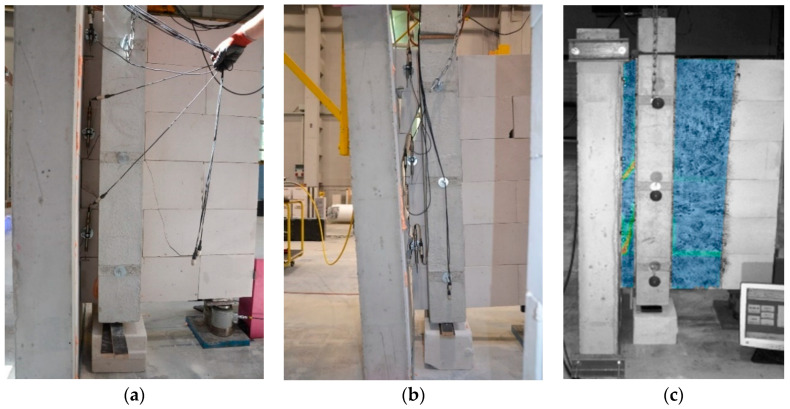
Experimental observations: (**a**) Visible diagonal/vertical cracks in wall C; (**b**) Failure of the joint between the tested walls; (**c**) Results of digital image correlation measurements [13].

All specimens showed multi-stage mechanism of failure. In the initial stage, the samples presented linear elastic response, no visible cracks were observed, and no cracking sounds were heard during this phase. At the end of the linear elastic response, a diagonal crack was observed in the lowest block that was located at the second row (Figure 4a,c). It was accompanied by the sound of cracking. Subsequently, additional cracks were noticed in the remaining two active blocks (Figure 4b). Further loading resulted in mutual displacement and rotation of the walls together with an increase of the existing cracks’ width. At the later stage of the loading, additional cracks were observed in the bed joints as well as crushing of the support zone of Wall B before the failure. In the post-cracking stage, which is defined as the stage after the appearance of first crack, the stiffness of the joint between the walls decreased significantly. After the sudden decrease of the measured force, the tests were stopped.

The summary of detailed results is shown in Table 1. The elastic stiffness of the joint between walls was calculated as follows:

(2) $$K_{el}=\frac{N_{0.6cr}}{u_{0.6cr}},$$

where *N_0.6cr_* is 60% of the load at initial cracking (*N_cr_*), and *u_0.6cr_* is the corresponding relative displacement. The assumption of level of the load (60% of *N_cr_*) to calculate elastic stiffness of the joint was an original idea of the authors. This is due to the fact that the development of cracking in masonry may proceed imperceptibly (e.g., internal damage to the wall) and first cracks invisible on the outer surface of the masonry units may form much earlier that visible cracks and sudden drop of force *N* being the basis for determining *N_cr_*. The assumption of the elastic stiffness of the connection *K_el_*, which is determined with a load of 0.6 × *N_cr_*, is also based on the observation of the load-displacement relationships shown in Figure 3. Up to this load level, almost linear load-displacement relationship is observed for all models. The load increase (in the range of 0.6 × *N_cr_*–*N_cr_*) causes a slight but visible weakening that reduces the stiffness of the joint.

In the initial (elastic) stage, the joint between the walls shows the average stiffness of 696 MN/m. First visible cracks were noticed at the average load of 39.5 kN and corresponding relative displacement of 0.09 mm. The failure occurred at the average load of 50.7 kN and corresponding relative displacement of 0.23 mm.

The relative variability (RV) of 23.1% was found for the load at first cracking. The large scatter can be explained by the fact that this phenomenon is characteristic for all brittle materials and may be related to the presence of non-homogeneously distributed flaws and microcracks in the material. Despite the relatively large scatter of the average load at initial cracking, the average value of the maximum load is more consistent (RV of 13.2%). In terms of the average values of the relative displacement at first cracking and at maximum load, RV was found to be 28% and 40%, respectively. Similarly, RV for the joint (elastic) stiffness was 20%, which is a consequence of the scatter of *N_0.6cr_* and the corresponding relative displacement *u_0.6cr_*.

## 3. Finite Element Modelling

Given the purpose of the current work, which was to simulate the phenomena occurring in the connection of two walls, it was decided to replace the existing wall (Figure 1d) with a micro-model that can be implemented in several ways. In the paper [42], it was stated that the micro-modelling of the wall can be carried out using the three-dimensional (3D) technique (Figure 1b) and the two-dimensional (2D) technique in a plane state of stress (less often in a plane state of deformation) shown in Figure 1c–f. For modelling connections, the authors abandoned the use of the 3D model and linear elastic models. The 2D micro-model shown in Figure 1d was used, in which AAC masonry elements AAC were given average mechanical parameters, and in masonry joints finite elements representing mortar were used. All elements were modelled in a plane state of stress.

### 3.1. Description of Model

Based on the experimental observations discussed in Section 2, the purpose of the current research contribution was focused on the structural behaviour of the joint between walls made of AAC masonry units under shear load. For the numerical analyses, the ABAQUS/Standard solver was used [43]. To ensure the computational efficiency and accuracy of numerical simulations, a 2D plane stress analysis and the so-called micro-modelling approach was adopted. The masonry units and mortar were modelled as continuum elements, and unit–mortar interfaces were tied together to account for a fully rigid connection.

**Figure 5:**
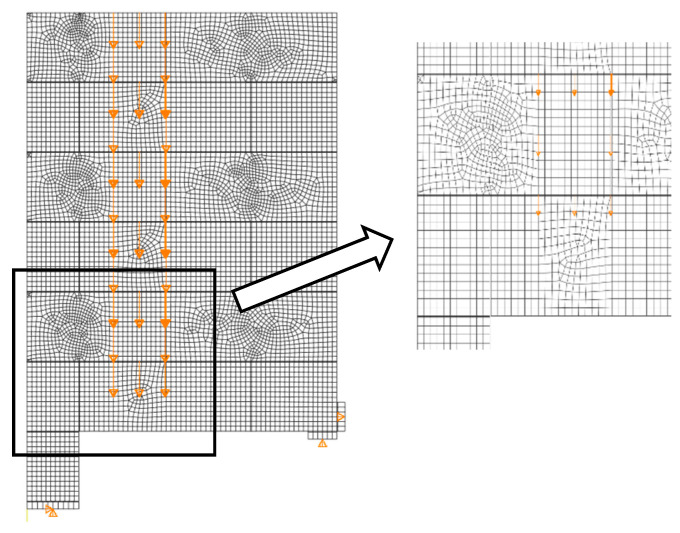
Overview of the 2D reference model (ABAQUS/Standard): Loads and boundary conditions and a detail of FE meshing.

The geometry and boundary conditions are shown in Figure 5. Under Wall A, a steel plate with the dimensions of 25 × 180 mm^2^ with a nodal support restraining the vertical and horizontal movements was modelled. Wall B was supported vertically and horizontally by steel plates 25 × 180 mm^2^ with appropriate supports that simulate the real boundary conditions during the tests. All nodal supports allowed for free in-plane rotation.

All elements were modelled using 4-node shell elements with reduced integration and large strain formulation (S4R type from the ABAQUS library [43]). A global mesh (15 mm) was applied to all elements while for mortar, due to its small thickness in relation to other components, three elements along its thickness were applied, which resulted in the element area of 1 × 3 mm^2^. The mesh was created by merging the nodes along the common boundaries of neighbouring regions into a single set of nodes. However, in some cases, tied surface interactions instead of merging these nodes were created to optimize the number of finite elements and decrease the computational time of the simulations. The meshing of the model is shown in Figure 5.

All calculations were run in displacement control including nonlinear effects of large deformations. The load was applied as a displacement (3 mm pointing downwards) distributed on the area where the vertical beams were located in the experiments (Figure 2). Although the T-shaped geometry of the joint, only three masonry elements from the B-wall made the joint. The face surfaces of these elements were influenced by the front surfaces of the masonry elements from wall A. Additional impacts of the constraining elements D resulted in a plane strain state in this area (ignoring the occurring friction). In the remaining layers, there was practically no contact between the front surfaces of the masonry elements from wall B and the facing surfaces of the elements of wall A. The connection of walls A and B, in which the joint was reduced to only three layers, enabled 2D modelling approach.

### 3.2. Materials

Careful consideration was paid to the mechanical characterisation of materials. The material data were taken from previous research contributions including mechanical characterisation of AAC units and mortar [44].

For steel, a linear elastic material with appropriate parameters was used [45]. The vertical joints (unfilled in the experiments) were filled with a very soft material with linear elastic formulation. Nonlinear behaviour of AAC and mortar is presented in Section 3.2.1. A summary of the material properties used in the study is presented in Table 2.

#### 3.2.1. Non-Linear Behaviour of AAC and Mortar

AAC and mortar were described by the concrete damage plasticity (CDP) material model available in ABAQUS/Standard software [43]. The CDP model is a continuum, plasticity-based damage model. It was originally implemented for concrete, however, it has been successfully applied to other brittle and quasi-brittle materials, such as masonry [46] and glass [47].

**Figure 6:**
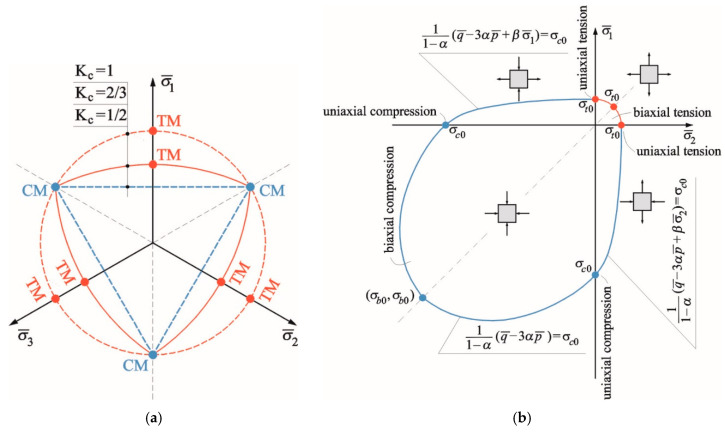
Yield surface in: (**a**) deviatoric plane; (**b**) plane stress. [43].

The model uses a concept of isotropic damaged elasticity in combination with isotropic tensile and compressive plasticity to represent the inelastic behaviour of a brittle material. In the CDP model, the yield surface function takes the form of an extended Drucker–Prager classical model and is based on the proposal of Lubliner [48], successively modified in accordance with [49] to take into account different evolution of strength under tensile and compressive stresses. The yield surface in the deviatoric conditions and plane stress and is shown in Figure 6a,b, respectively.

The CDP model assumes non-associated potential plastic flow. The flow potential G used for this model is the Drucker–Prager hyperbolic function according to the formula:

(3) $$G=\sqrt{{\left(\varepsilon \sigma_{t0}\tan\phi \right)}^{2}+{\overset{¯}{q}}^{2}}-\overset{¯}{p}\tan\phi ,$$

where φ is the is the dilatation angle measured in the *p* − *q* plane at high confining pressure, $\sigma_{t0}$ is the uniaxial tensile stress at failure (taken from the user-specified tension stiffening) data, and ϵ is the parameter (referred to as the eccentricity) that defines the rate at which the function approaches the asymptote, and sigma is the uniaxial tensile stress at failure.

In the plane stress state, the failure criterion was described by four equations:

- in the first quarter of the coordinate system, the criterion is described by the equation of a circle with a radius equal to the uniaxial tensile strength *f_t_*:
  (4) $$f\left(\sigma_{c0},\kappa \right)=\frac{1}{1-\alpha }\left(\bar{q}-3\alpha \bar{p}+\beta \bar{\sigma_{1}}\right)-\sigma_{c0}=0,$$
- in the third quarter of the coordinate system:
  (5) $$f\left(\sigma_{c0},\kappa \right)=\frac{1}{1-\alpha }\left(\bar{q}-3\alpha p\right)-\sigma_{c0}=0,$$
- in the third quarter of the coordinate system:
  (6) $$f\left(\sigma_{c0},\kappa \right)=\frac{1}{1-\alpha }\left(\bar{q}-3\alpha \bar{p}+\beta {\bar{\sigma }}_{2}\right)-\sigma_{c0}=0,$$
  (7) $$\alpha =\frac{\sigma_{b0}-\sigma_{c0}}{2\sigma_{b0}-\sigma_{c0}},$$
  (8) $$\beta \left(\kappa \right)=\frac{\sigma_{c0}}{\sigma_{t0}}\left(1-\alpha \right)-\left(1+\alpha \right),$$
  where $\sigma_{b0}$ is the material strength in biaxial compression, $\sigma_{c0}$ is the tensile strength of a material, *α*, *β* are the model parameters, α is the parameter determined on the basis of the initial values of yield strength in uniaxial and biaxial compression, *β* is the parameter dependent on the hardening variable *κ*, determined on the basis of the proportion of the initial values of uniaxial yield strengths in compression and tension.

In the Formulas (6)–(9), *p* and *q* are the stress tensor invariants, *κ* is the stiffening variable, expressed by two independent stiffening variables, respectively, for tension and compression, α is the model parameter that is determined on the basis of initial values of yield stress in uniaxial and biaxial compression, *β* is the model parameter that is dependent on the variable *κ*, determined on the basis of the proportion of initial values of uniaxial yield stress in compression and tension.

The CDP model requires defining the plasticity limits of the material under uniaxial compression, biaxial compression and uniaxial tension (parameters responsible for the shape of the plasticity surface) as well as the expansion angle and the *ε* coefficient that determine the shape of the surface of the plastic potential necessary to assume the non-associated flow law.

**Figure 7:**
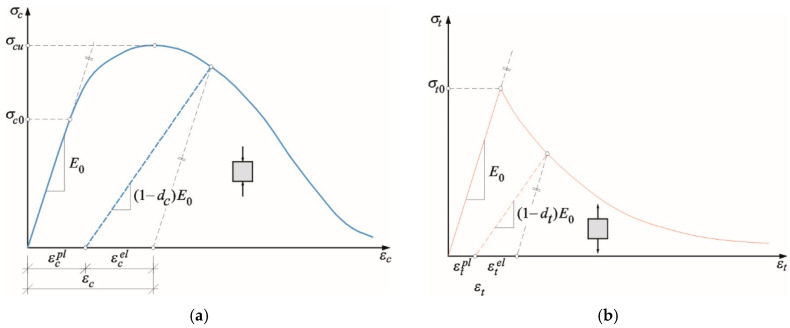
Mechanical constitutive law for the CDP model in: (**a**) compression; (**b**) tension [43].

The model assumes that the uniaxial tensile and compressive response of concrete is characterised by damaged plasticity. It describes the inelastic compressive and tensile behaviours of a given brittle material in the form of multi-hardening plasticity and scalar isotropic damaged elasticity characteristic curves (Figure 7). Under uniaxial compression, the response of the material is linear until the value of initial yield strength *σ_c0_*. In the plastic regime, the response is typically characterised by stress hardening followed by strain softening beyond the maximum stress *σ_c_*_u_. This representation, although simplified, captures the main features of the response of a brittle material. For tensile behaviour, Hillerborg’s [50] fracture energy proposal is applied (Figure 7a). It defines the energy required to open a unit area of crack, as a material parameter, using brittle fracture concepts. With this approach, the material’s brittle behaviour is characterised by a stress–displacement response rather than a stress–strain response. The fracture energy is specified directly as a material property. In this case, the property is defined by the failure stress and function of the associated fracture energy. This model assumes a linear loss of strength after cracking, as shown in Figure 7a. The cracking displacement at which complete loss of strength takes place (*u_t_*_0_) is, therefore,

(9) $$u_{t0}=\frac{2G_{f}^{I}}{\sigma_{t0}},$$

where $G_{f}^{I}$ is the fracture energy and *σ_t0_* is the tensile strength of a material.

As presented in Figure 6, when the specimen is unloaded from any point on the strain-softening branch of the stress–strain curves, the unloading response is weakened: the elastic stiffness of the material is damaged. The degradation of the elastic stiffness is characterised by two damage variables *d_t_* and *d_c_* for tension and compression behaviour, respectively. The damage variables can take values from zero, representing the undamaged material, to one, which represents total loss of strength. If *E*_0_ is the initial (undamaged) elastic stiffness of the material, the stress–strain relations under uniaxial tension and compression loading are, respectively:

(10) $$\sigma_{t}=\left(1-d_{t}\right)E_{0}\left(\varepsilon_{t}-\varepsilon_{t}^{pl}\right)$$

(11) $$\sigma_{c}=\left(1-d_{c}\right)E_{0}\left(\varepsilon_{c}-\varepsilon_{c}^{pl}\right)$$

In the analyses, it was assumed that the damage variables *dt* and *dc* are changing linearly from 0.0 to 0.8 at the maximum value of the inelastic strain. The default values of parameters used in the model are adopted in the same way as in case of brittle materials such as concrete according to [43,46,47,48].

The values used in this study were based on the default values for the CDP model literature references [43], and they are summarised in Table 3.

#### 3.2.2. Compressive and Tensile Behaviour of AAC and Mortar

**Figure 8:**
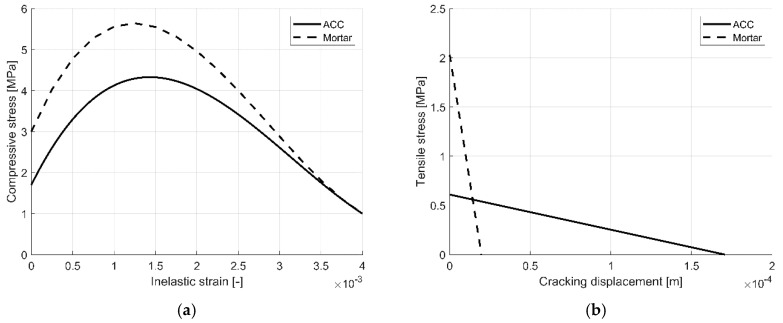
Material model of AAC and mortar [44]: (**a**) Compressive stress–inelastic strain curves (compressive behaviour); (**b**) Tensile stress–cracking displacement (tensile behaviour).

The behaviour of AAC and cement mortar (in compression and tension) used in the numerical simulations is shown in Figure 8. The figures present the results of previous experimental investigations reported in [44]. The response of AAC and cement mortar under uniaxial compression is linear until the value of initial yield, *σ*_c0_. In the plastic regime, the response is typically characterised by stress hardening, followed by strain softening beyond the maximum stress *σ*_cu_. It captures the main features of the response of brittle material. The response of AAC and cement mortar under uniaxial tension is that the stress–strain response follows a linear elastic relationship until the value of the failure stress *σ_t_*_0_ is reached. The failure stress corresponds to the onset of microcracking in the material. Beyond the failure stress, the formation of microcracks is represented macroscopically with a softening stress–strain response, which induces strain localisation in the structure.

## 4. Finite Element Results and Discussion

### 4.1. Verification of the Reference FE Model

**Figure 9:**
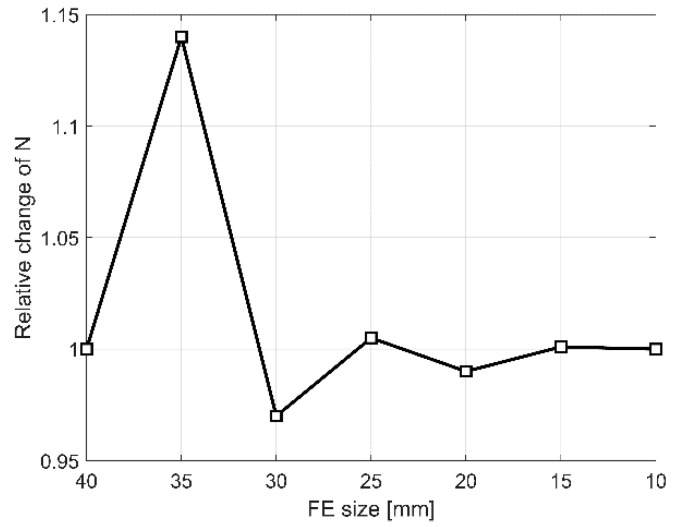
Convergence of the numerical model in terms of relative change of maximum load.

The first step of validation of the reference FE model was a convergence study (Figure 9). Its aim was to verify the mesh quality and investigate how the reference FE model converges on the true solution depending on the FE size of AAC units.

The FE mesh, presented in Figure 5, was studied, and different FE sizes for AAC units were investigated. The study covered the variation of FE size from 40 to 10 mm, which corresponds to 16.7% and 4.2% of the AAC unit height, respectively. The results of the convergence studies were processed by examining the relative change in output variable extrema, in this case the maximum load.

Figure 9 shows the relationship of the relative change (in relation to the previous step) of the maximum load to the FE size of AAC units. From the study, it was concluded that the FE mesh converges to a sufficient degree at 15 mm, and thus this FE size was kept throughout all further analyses.

The FE model was then validated using the experimental results. Table 4 summarises the comparison of experimental and numerical results in terms of the load at initial cracking and the maximum load together with the corresponding relative displacements and the joint stiffness. For the purpose of the validation, the experimental results were considered as reference values.

**Figure 10:**
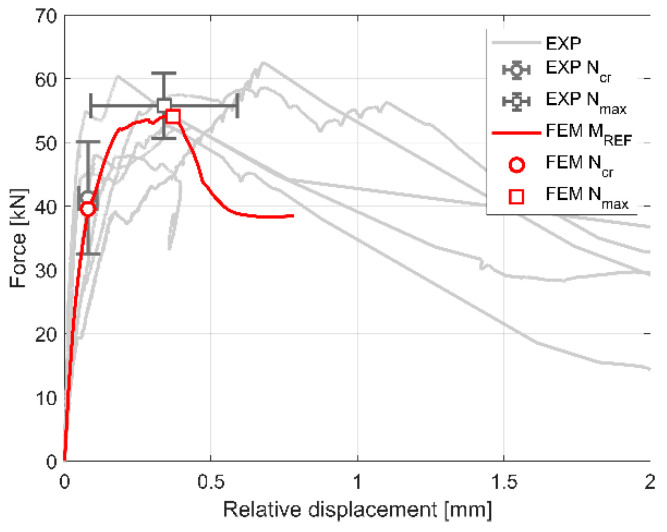
Experimental and numerical (reference model) load–relative displacement responses of the walls.

Table 4 shows that the values of *N_cr,FEM_*/*N_cr,EXP_* and *u_cr,FEM_*/*u_cr,EXP_* are 1.01 and 0.92, respectively. It indicates that the FE model is capable of predicting the load at initial cracking and the corresponding relative displacement between the walls with a reasonably good accuracy (less than 10%). In terms of the maximal load and the corresponding relative displacement, *N_max,FEM_*/*N_max,EXP_* and *u_max,FEM_*/*u_max,EXP_* are 0.92 and 1.64, respectively. Regarding the joint stiffness in the elastic stage, the numerical model overestimates the test results by 13%. The higher stiffness of the numerical model can be explained by the fact that the model does not take into account potential movements, slips and deformations in the test set-up, which is a common observation. Similar differences between the results of the experiments and simulations (in terms of displacements in the elastic phase) were also found in [19,23,33,50]. Experimental and FE load vs. displacement curves are plotted in Figure 10. In the figure, experimental results are shown by grey lines, whereas the FE curve is plotted with a red line. The average experimental values of the load at initial cracking (EXP *N_cr_*) and at maximum load (EXP *N_max_*) with error bars corresponding to the coefficients of variations of the experimental results are presented. The values correspond to the experimental results shown in Table 1. In Figure 5, values of the load at initial cracking (FEM *N_cr_*) and at maximum load (FEM *N_max_*) together with corresponding displacements obtained from numerical studies are presented.

The FE curves, in general, compare reasonably well with the experimental results. The model is capable of capturing the initial stiffness degradation of the AAC elements and cracking development until the maximum load. It is also able to produce reasonable results of the stiffness softening after this stage. At the relative displacement of approximately 0.8 mm, the simulation stopped converging due to the excessive distortion of the FE. However, this stage of the joint’s behaviour is less important for the analyses because the main aim of the study was to simulate numerically the behaviour of the model until maximum load which is critical for structural considerations.

**Figure 11:**
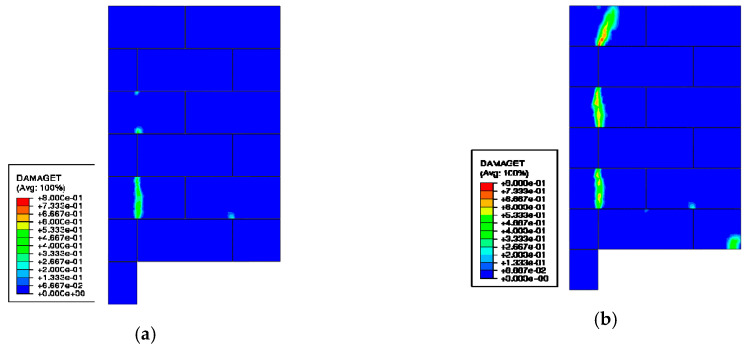
Map of tensile damage of AAC units at: (**a**) initial cracking; (**b**) maximum load.

Figure 11 shows the cracking patterns obtained from the FE model at initial cracking and the maximum load. Besides the cracking at the three AAC units of Wall A actively transferring shear stress, local failure close to the support zone of Wall B was also found. This corresponds well to the experimental observation in which the support zone was also prone to failure at the later stage of loading.

### 4.2. Sensitivity Analysis

The validation of the model showed that the FE model is effective and accurate in simulating the load–relative displacement responses and cracking pattern of the traditional joints between walls made of AAC masonry units. However, input material parameters for AAC assumed in the study were based on the average values found in the experimental campaign [44]. It was important to investigate the influence of the range of variation of these parameters (found in experimental studies reported in [15]) on the FE results. Table 5 presents the overview of models that were investigated.

From the sensitivity studies, it was found that variation of elastic modulus, tensile strength and fracture energy affect different output results (Table 6). It was also found that variation of Poisson’s ratio and compressive strength has negligible effect (less than 2%) on the results.

**Figure 12:**
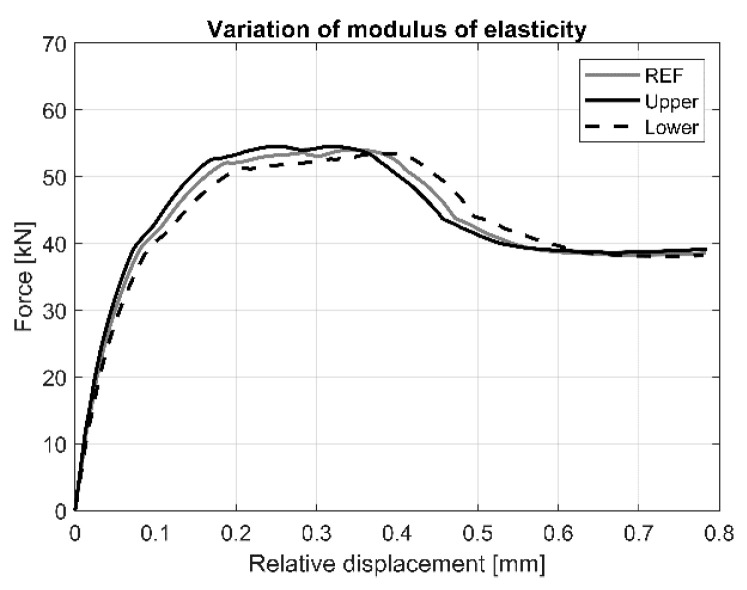
Load vs. load-relative displacement curves for the variation of elastic modulus of AAC.

The effect of the variation of the elastic modulus on the load vs. relative displacement response is shown in Figure 12. Results indicate that this parameter has a significant effect on initial stiffness, the relative displacement at the initial and maximum load, whereas it has negligible effect on the value of the load at initial cracking and maximum load.

**Figure 13:**
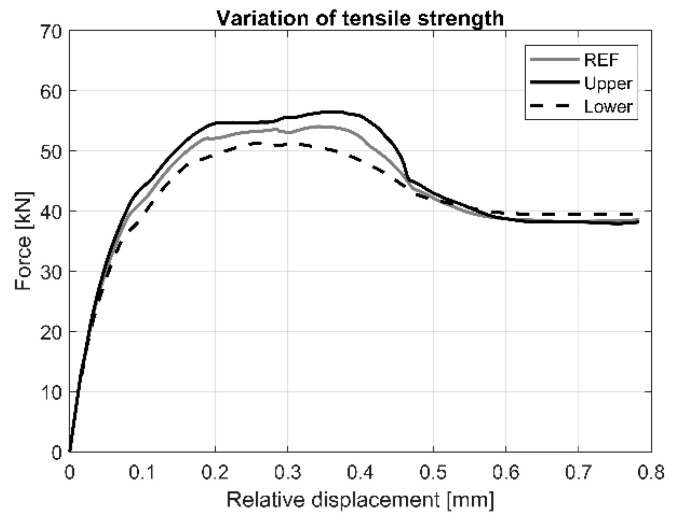
Load vs. load-relative displacement curves for the variation of tensile strength of AAC.

The effect of the variation of tensile strength on the load vs. relative displacement response is shown in Figure 13. Results indicate that this parameter has a significant effect on load at first cracking, maximum load, the relative displacement at the initial and at the maximum load. As expected, it has no effect on the initial stiffness.

**Figure 14:**
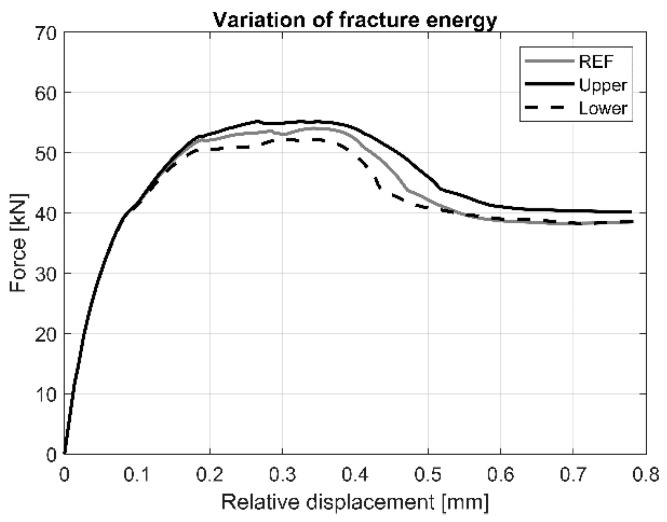
Load vs. load-relative displacement curves for the variation of fracture energy of AAC.

The effect of the variation of fracture energy on the load vs. relative displacement response is shown in Figure 14. Results indicate that this parameter has only effect on the maximum load and the behaviour at the post-elastic stage. Higher value of the fracture energy allows for larger deformation.

## 5. Conclusions

In this paper, a nonlinear two-dimensional finite element model was developed to simulate the structural behaviour of traditional joints between walls made of AAC masonry units. The finite element model was validated using the test results of an experimental campaign involving testing of six T-shaped, monosymmetric samples with traditional joints between walls loaded in shear. A sensitivity study of several critical input material parameters of AAC on the behaviour of the walls was also conducted. From the performed studies, the following conclusions are drawn:

- The 2D nonlinear model developed is capable of producing accurate results and predicting the load at initial cracking and the maximum load together with corresponding relative displacements between the walls with a reasonably good accuracy.
- From the sensitivity studies, it was found that the variation of elastic modulus, tensile strength and fracture energy of AAC is most critical to the results of the simulations (FE curve). It was also found that variation of Poisson’s ratio and compressive strength has negligible effect on the results.

Further comprehensive tests should aim at understanding the mechanism of cracking and failure of AAC wall joints and also comparing the load-bearing capacity of wall joints made with traditional bonding techniques and various types of fasteners.

## Figures and Tables

**Table 1:** Experimental results for the tested specimens [13].

Sample	Load at First Cracking	Maximum Load	Relative Displacement at First Cracking	Relative Displacement at Maximum Load	Joint (Elastic) Stiffness (2)
	*N_cr_* [kN]	*N_max_* [kN]	*u_cr_* [mm]	*u_max_* [mm]	*K_el_* [MN/m]
P-1	27.3	56.3	0.07	0.31	625
P-2	42.6	50.0	0.12	0.25	625
P-3	31.2	38.6	0.12	0.16	588
P-4	54.1	59.8	0.07	0.17	625
P-5	35.1	48.1	0.06	0.10	714
P-6	45.1	51.6	0.08	0.36	1000
Average	39.5 ± 9.0	50.7 ± 6.7	0.09 ± 0.02	0.23 ± 0.09	696 ± 141

**Table 2:** Summary of material properties for test specimens [30,44,45].

Material	Density [kN/m^3^]	Elastic Modulus [MPa]	Poisson’s Ratio [-]	Compressive Strength [MPa]	Tensile Strength [MPa]	Fracture Energy *G_f_^I^* [N/m]
AAC Mortar	0.25 16.0	28,860 6,351	0.20 0.18	4.25 5.64	0.61 2.03	52.152 20.0
Steel	78.5	210,000	0.30	-	-	-
Gap infill	10	1	0.45	-	-	-

**Table 3:** The CDP model parameters for AAC and mortar. [43].

Dilatation Angle *Ψ_c_*	Eccentricity ϵ	*σ_b0/_σc_0_*	*K_c_*	Viscosity
35	0.1	1.16	0.667	0

**Table 4:** Experimental results for the tested specimens [13] and numerical FE predictions (ABAQUS/Standard).

Sample	Load at First Cracking	Maximum Load	Relative Displacement at First Cracking	Relative Displacement at Maximum Load	Joint Stiffness
	*N_cr_* [kN]	*N_max_* [kN]	*U_cr_* [mm]	*u_max_* [mm]	*K_el_* [MN/m]
EXP (average)	39.5	50.7	0.09	0.23	696
FEM	39.5	54.0	0.08	0.37	788
FEM/EXP	1.01	1.07	0.92	1.64	1.13

**Table 5:** Summary of input parameters of AAC used in the sensitivity study [44].

	Elastic Modulus [MPa]	Poisson’s Ratio [-]	Compressive Strength [MPa]	Tensile Strength [MPa]	Fracture Energy [N/m]
Variation	±10.5%	±8.5%	±7.3%	±14.0%	±15.0%

**Table 6:** Results of sensitivity studies.

Model	Load at First Cracking	Maximum Load	Relative Displacement at First Cracking	Relative Displacement at Maximum Load	Joint Stiffness
	*N_cr_* [kN]	*N_max_* [kN]	*u_cr_* [mm]	*u_max_* [mm]	*K_el_* [MN/m]
M_REF_	39.55	54.04	0.08	0.37	788.46
M_MOE,Upper_	39.55 (1.01)	54.56 (1.01)	0.07 (0.88)	0.32 (0.86)	909.09 (1.15)
M_MOE,Lower_	39.49 (1.00)	53.46 (0.99)	0.09 (1.13)	0.39 (1.05)	714.29 (0.91)
M_FT,Upper_	44.01 (1.11)	56.54 (1.05)	0.10 (1.25)	0.37 (0.92)	788.46 (1.00)
M_FT,Lower_	35.59 (0.90)	51.14 (0.95)	0.07 (0.88)	0.31 (0.84)	788.46 (1.00)
M_GF,Upper_	39.55 (1.00)	55.19 (1.02)	0.08 (1.00)	0.37 (1.00)	788.46 (1.00)
M_GF,Lower_	39.55 (1.00)	52.23 (0.97)	0.08 (1.00)	0.31 (0.84)	788.46 (1.00)
